# Checklist Approach to Developing and Implementing AI in Clinical Settings: Instrument Development Study

**DOI:** 10.2196/65565

**Published:** 2025-02-20

**Authors:** Ayomide Owoyemi, Joanne Osuchukwu, Megan E Salwei, Andrew Boyd

**Affiliations:** 1 Department of Biomedical and Health Informatics, University of Illinois Chicago, 1919 W Taylor, Chicago, IL, 60612, United States, 1 3129782703; 2 College of Medicine, University of Cincinnati, Cincinnati, OH, United States; 3 Department of Biomedical Informatics, Vanderbilt University Medical Center, Nashville, TN, United States

**Keywords:** artificial intelligence, machine learning, algorithm, model, analytics, AI deployment, human-AI interaction, AI integration, checklist, clinical workflow, clinical setting, literature review

## Abstract

**Background:**

The integration of artificial intelligence (AI) in health care settings demands a nuanced approach that considers both technical performance and sociotechnical factors.

**Objective:**

This study aimed to develop a checklist that addresses the sociotechnical aspects of AI deployment in health care and provides a structured, holistic guide for teams involved in the life cycle of AI systems.

**Methods:**

A literature synthesis identified 20 relevant studies, forming the foundation for the Clinical AI Sociotechnical Framework checklist. A modified Delphi study was then conducted with 35 global health care professionals. Participants assessed the checklist’s relevance across 4 stages: “Planning,” “Design,” “Development,” and “Proposed Implementation.” A consensus threshold of 80% was established for each item. IQRs and Cronbach α were calculated to assess agreement and reliability.

**Results:**

The initial checklist had 45 questions. Following participant feedback, the checklist was refined to 34 items, and a final round saw 100% consensus on all items (mean score >0.8, IQR 0). Based on the outcome of the Delphi study, a final checklist was outlined, with 1 more question added to make 35 questions in total.

**Conclusions:**

The Clinical AI Sociotechnical Framework checklist provides a comprehensive, structured approach to developing and implementing AI in clinical settings, addressing technical and social factors critical for adoption and success. This checklist is a practical tool that aligns AI development with real-world clinical needs, aiming to enhance patient outcomes and integrate smoothly into health care workflows.

## Introduction

The implementation of any technology in a real-world setting, especially a clinical one, requires adequate consideration of the social aspects of its application alongside the technical considerations [1]. The National Academy of Medicine report highlighted the need to “understand the technical, cognitive, social, and political factors in play and incentives impacting integration of Artificial Intelligence (AI) into health care workflows” [2]. It is important to understand the context in which the technology will be used, how it will work with existing workflows without disruption, and how it will be accepted by the people who will have to use it. Historically, in the development of AI systems, the technical perspective has taken preeminence over how they fit and work in the real world, and this has resulted in AI systems falling short of their translational goals [3]. In general, AI tools have shown promise in development, but few have been able to translate into the real-world settings for patient management [4]. For example, for a management decision tool built and deployed in a hospital in Utah for diabetes management, there was a challenge of not offering all the information that was desired by clinicians and patients to decide on type 2 diabetes management [5].

Despite the numerous proof-of-concept publications in this field, the lack of robust frameworks for supporting the development and management of these tools has been one of the main barriers to their adoption in health care [6]. There is a paucity of specific guidance and rigorous best practices for people designing and developing AI solutions targeted at clinical settings and use cases. A review conducted by Gama et al [7] highlighted the need to develop an AI-specific implementation framework because there is an unrealized opportunity to draw insights from implementation science, as well as to use theoretical and practical insights, to accelerate and improve on the implementation of AI in clinical settings.

There have been a few frameworks and guidelines proposed recently. Salwei and Carayon [1] developed a sociotechnical systems framework for AI that acknowledges the social and technical aspects of work that relate to the successful design and implementation of AI. Their model demonstrates that an AI can only integrate into clinical workflows if it fits within the context, or the work system, in which it is implemented. The CONSORT (Consolidated Standards of Reporting Trials)-AI extension and TRIPOD (Transparent Reporting of a Multivariable Prediction Model for Individual Prognosis or Diagnosis) are examples of models that are narrow in their application and are focused on trials, performance, and comparison, which are only helpful in a single phase of the AI life cycle [8,9]. However, most of the existing frameworks gloss over relevant sociotechnical factors, while others only target specific stages in the AI development cycle, and almost all have no easy-to-use checklist. This study sought to develop a framework and operationalize it as a checklist that covers all the aspects of the development cycle and holistically addresses sociotechnical factors across those phases.

## Methods

### Literature Synthesis

We conducted a literature search on the MEDLINE via OVID and Embase databases between June 25 and 30, 2023. Our search focused on studies examining AI in clinical settings, particularly those addressing frameworks, guidelines, and theories for AI implementation, design, and evaluation. The following keywords were used in the search: “Artificial intelligence,” “Framework,” “Guideline,” “Theory,” “Implementation,” “Evaluation,” “Design,” “Development,” “Clinical Settings,” “Clinical Care,” “Hospital,” “Clinic,” and “Patient Care.” There were no restrictions on the publication dates of the studies, meaning articles from any year were considered in the search. This initial search identified 573 potential studies. We screened the abstracts of these studies using the following inclusion criteria:

- Studies involving the application of AI by health care providers in a clinical setting
- Research that used a conceptual or theoretical framework related to AI in clinical care
- Primary qualitative studies that focused on the design, implementation, or evaluation of AI in clinical care, regardless of whether a distinct framework was used

We excluded studies that:

- Focused primarily on patient-related outcomes
- Concentrated on the technical or computational aspects of AI without clinical integration

We identified 19 relevant studies for full-text review. Three were excluded (one reporting guideline, one study protocol, and one commentary). Through citation tracking, we added 4 additional relevant studies, bringing the final sample to 20 articles. These 20 studies were thoroughly reviewed, and key points, themes, and insights were extracted. We then synthesized these insights with findings from a previously conducted primary study [10] on the implementation and user experience of an AI-powered sepsis alert system. Using a mind map approach, we organized the themes and insights into key domains to develop our framework.

### The Modified Delphi Study

The framework developed from the literature synthesis was used to develop a preliminary draft of a checklist targeted at supporting teams designing and developing AI systems for clinical settings. This draft was shared with selected experts for review, edits, and improvements using a Delphi method. The Delphi method is a procedure for reaching a consensus with a group of people who are typically experts on the subject through controlled assessments [11]. The technique has been used in health care to achieve consensus in establishing guidelines or treatment protocols when evidence is limited, inadequate, or contradictory [12]. For this study, a modified approach was used, which involved the development of the initial checklist questions by the researcher rather than the panelists. This approach ensured that the questions were grounded in the literature framework and leveraged the researcher’s expertise. This modification helped streamline the process and ensure that the questions were relevant to the specific context of AI system development in clinical settings. The panelists were then asked to refine and validate these questions, rather than generating them from scratch.

The modified Delphi study was conducted between January 23 and March 14, 2024. The selection of Delphi panelists followed a process aimed at ensuring diversity in expertise and professional background. Potential participants were recruited through targeted outreach on platforms such as email listserves, LinkedIn, Twitter, and closed WhatsApp groups. To be eligible, participants were required to hold advanced degrees and have at least 2 years of professional experience in fields directly related to AI systems in health care. Specifically, panelists were selected based on their expertise in areas such as medicine (doctors and nurses), health informatics, AI research, AI engineering, health care administration, human factors research, health care system research, implementation science, health care product management, health ethics, and safety. The global nature of the study welcomed participants from any country, ensuring a broad range of perspectives.

Interested individuals were initially asked to complete a preliminary form to provide background information about their experience and qualifications. This form was used to filter suitable candidates for inclusion in the Delphi panel. Invitations were then sent to selected candidates, along with a detailed information letter explaining the study’s goals and procedures. A pretest was conducted with a panel comprising 5 professionals, each with some expertise in the fields of health care and technology. Their feedback helped refine the checklist to ensure clarity, making it easier for participants to understand and respond accurately.

Participants who agreed to take part accessed the first round of the Delphi survey through a link in the email, which led to the consent form and survey. Data collection was done using Google Forms. To avoid bias, the panelists remained anonymous to each other throughout the process.

The preliminary survey comprised 45 questions designed to assess the relevance of each checklist item to the AI system’s design and development process. A Likert scale from 1 (“Not Relevant”) to 5 (“Highly Relevant”) was used, along with open-ended comment fields for feedback and suggestions. The checklist was organized into four stages of AI system development: (1) planning, (2) design, (3) development, and (4) proposed implementation. Each stage aligned with 1 of the 6 domains in our framework.

**Figure 1.**
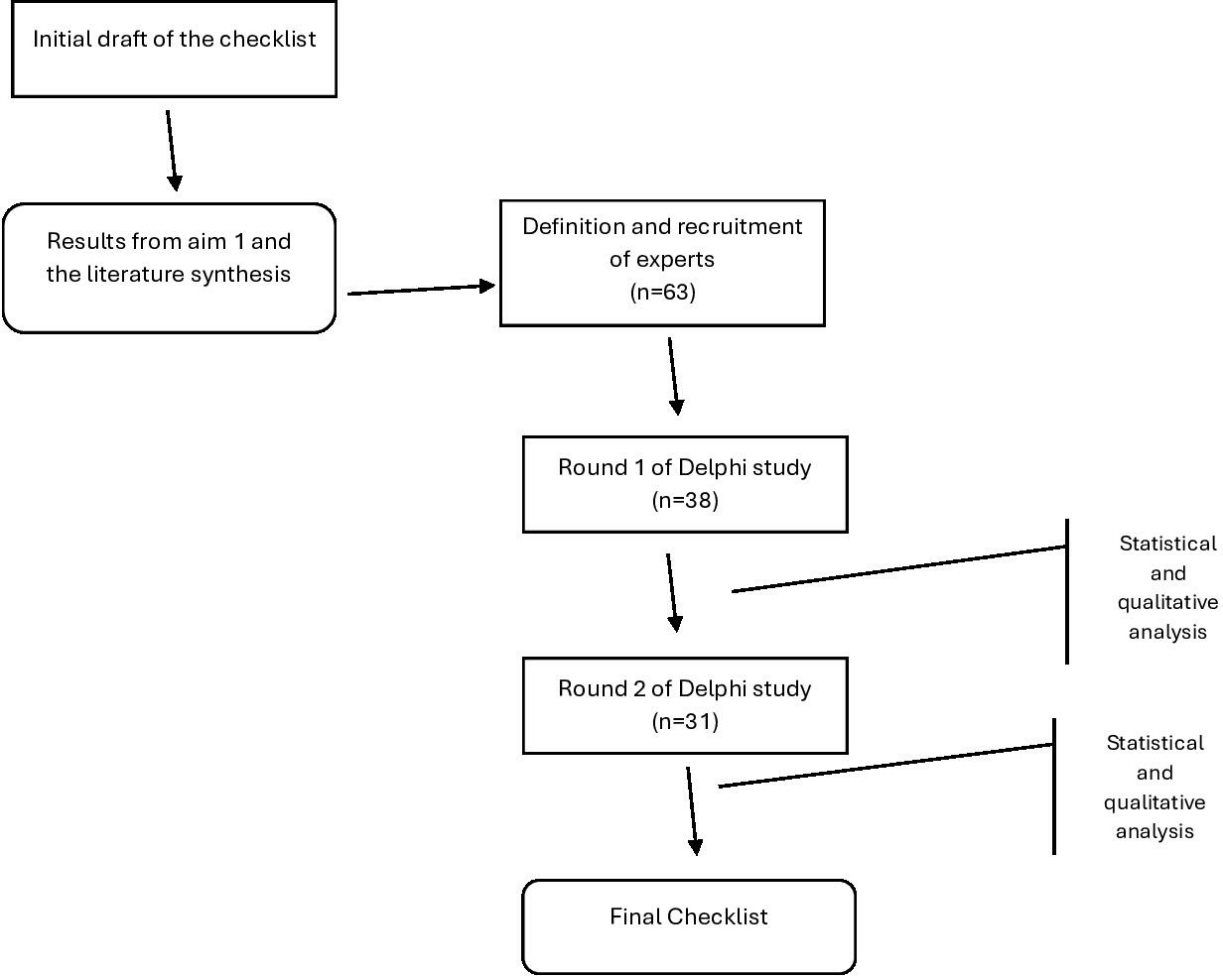
The process of developing the checklist.

After completion of the preliminary survey, the results were analyzed to assess the level of consensus among panelists. Based on the analysis, along with participants’ feedback and comments, the checklist was revised and updated for the second round of the Delphi process. All the initial panelists were also invited for the second round even if they missed the first. This approach was based on the study by Boel et al [13], which showed that inviting panel members who missed a previous round to a subsequent round led to better representations of opinions and reduced the chances of false consensus while not influencing the outcome. The results of the analysis and feedback were added to the questionnaire for the second round. The whole process is highlighted in Figure 1.

Questions rated 4 or higher were classified as “relevant” to streamline the analysis. At the same time, those rated 3 or lower were deemed “irrelevant.” This categorization facilitated a more efficient evaluation of the panelists’ responses. Descriptive statistics were used to analyze the results of each round, along with an analysis of the IQR for each question. In determining the threshold for consensus among panelists, a mean score of 0.8 (representing 80% agreement) was established a priori as the benchmark. Questions with a mean score above 0.8 and an IQR of 0 were deemed to have consensus among the participants. Lastly, the Cronbach α reliability coefficient was calculated to evaluate the interitem reliability. The qualitative data collected during each round were analyzed using inductive content analysis. Quantitative analyses were conducted using the Python programming language in JupyterLab for Windows (Project Jupyter).

### Ethical Considerations

This study was conducted in accordance with institutional ethical guidelines for research involving human subjects and was approved by the University of Illinois Chicago Institutional Review Board under protocol STUDY2023-0535-MOD003. Participants provided informed consent, ensuring they were aware of the study’s purpose, procedures, potential risks, and their right to withdraw at any time. All data collected were either anonymized or deidentified to protect participant privacy, with strict safeguards in place to ensure confidentiality. Additionally, no financial or material compensation was provided to participants in this Delphi study, and participation was entirely voluntary.

## Results

### Literature Synthesis

**Figure 2.**
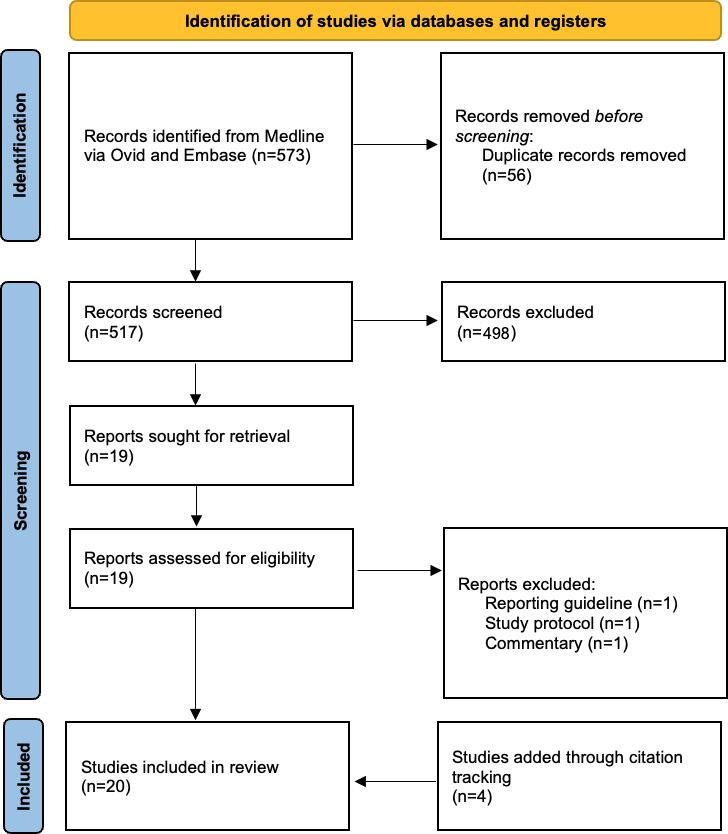
PRISMA (Preferred Reporting Items for Systematic Reviews for Meta-Analyses) flowchart.

**Figure 3.**
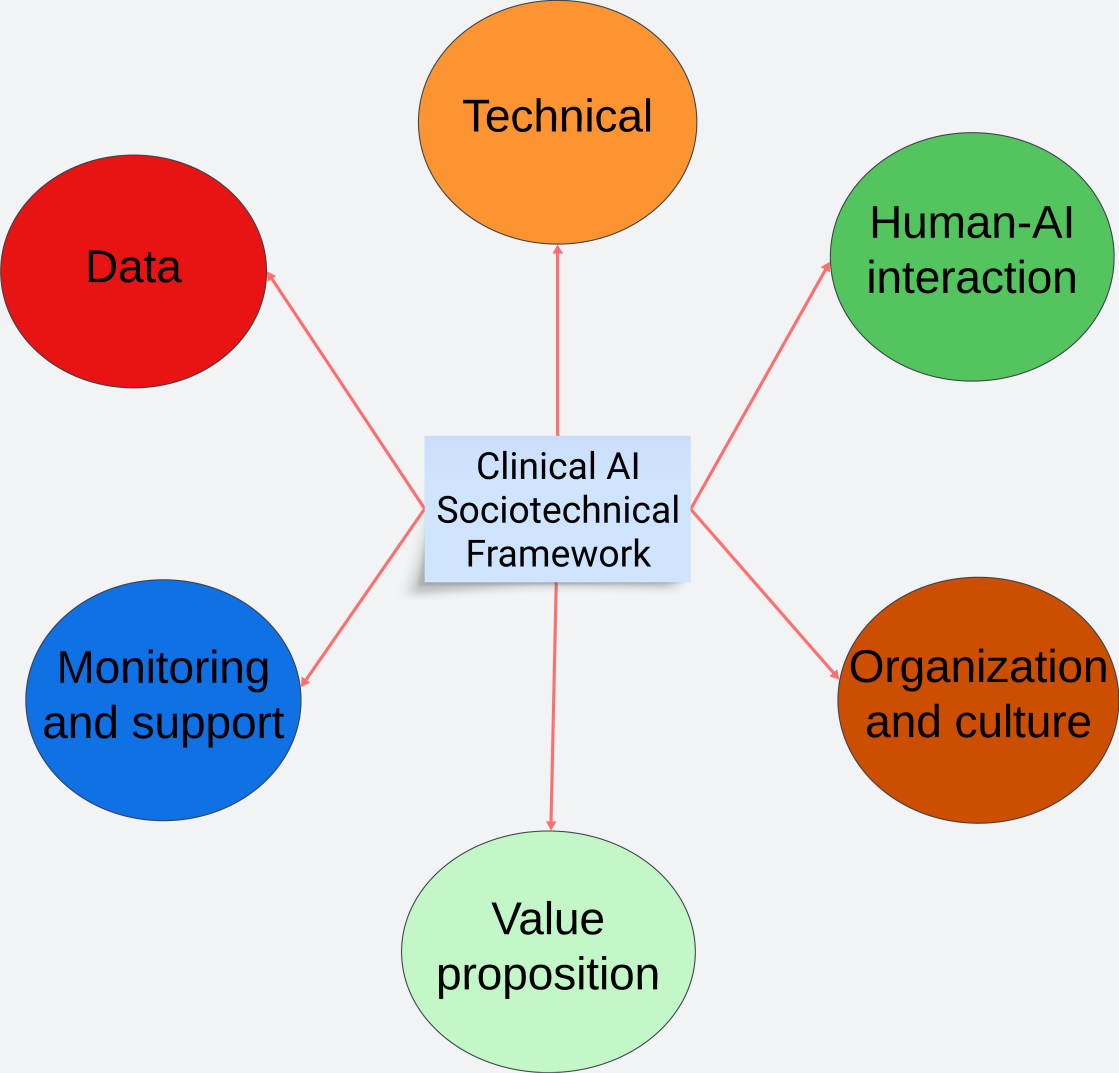
The Clinical AI Sociotechnical Framework. AI: artificial intelligence.

The literature search identified 20 studies [1,3,7,14-30] that proposed a framework, guideline, or approach for the design, development, implementation, or evaluation of AI for clinical use cases (Figure 2). A total of 14 (65%) of these addressed specific areas in the AI development cycle, from design to maintenance and management, while some cut across every aspect of the cycle. The results of the literature search were synthesized with the primary research and connected using a mind map to arrive at the domains of the Clinical AI Sociotechnical Framework (CASoF), which is a sociotechnical framework to support the planning, design, development, and proposed implementation of AI systems to help better plan and predict the likely success of the AI system (Figure 3).

### The Modified Delphi Study

Based on the CASoF, the first draft of the checklist was developed, which was shared with a team of panelists for evaluation and review using a Delphi approach. A total of 65 panelists were recruited: 21 (32%) doctors, 10 (15%) health care experts or researchers, 9 (12%) AI researchers, 4 (6%) health informaticians, 4 (6%) nurses, and 18 (28%) other professionals. Of the 65 panelists invited to participate in the study, 35 (54%) of them completed the first round of Delphi. The initial checklist had 4 overall categories that corresponded to the 4 stages in the development and deployment process, with 15 subcategories that corresponded to the domains of the CASoF that were important in each of the stages. The stages were “Planning,” “Design,” “Development,” and “Proposed Implementation.” As part of the questionnaire, panelists were asked 2 open-ended questions at the end of each of the subcategories: “Would you reframe any of the questions above?” and “Are there questions that you would add or remove from this segment?” During the first round of the Delphi, panelists suggested multiple edits and additions to the checklist. This suggested editing included the need to reframe some of the questions to make them more appropriate and clearer for a checklist. In one of the subcategories, one panelist responded as follows:

The last question says, “data processing.” That comes across as ambiguous. What does that refer to? who will be the audience for this survey? will they understand what that means? Are we trying to abstract curation, cleaning etc into abstraction?

At the end of the survey, panelists were asked why they might not use the checklist, and some of the responses included the following:

I think the checklist is long. The challenge when you have checklists this long is that people tend to gloss over them and are not intentional about answering the questions in a detailed way.

Might be helpful to shorted and make more actionable. eg, policies and procedures document has been completed versus have you considered a place for policies.

The checklist is somewhat burdensome on the AI vendor and health system. I would cut the questions in half.

These open-ended questions were analyzed using a content analysis approach to bring out the recurrent themes and perspectives shared by the panelists in reforming and improving the questionnaire. Quantitative analyses were done, which showed a high level of agreement and relevance across most questions. Descriptive analysis was done: the mean score for the relevance of the questions on the survey exceeded 0.8 on all but one, indicating that at least 80% of respondents found the questions pertinent to their work and the topic at hand. Furthermore, the IQR was calculated to be 0 for all questions except 3, highlighting a level of consensus among respondents. The consensus and the structure of the checklist are shown in Multimedia Appendix 1.

Based on the results, comments, and feedback from the panelists, the checklist was revised. The “Design” and “Development” stages were merged into a single stage, and the “People” and “Organization and Culture” domains were merged into a single domain. The “User Experience and Workflow” and “Clinical Utility” domains were merged to create a new domain called “Human-AI Interaction.” The total number of questions was reduced from 45 questions to 34 questions to make it less cumbersome and more focused. These 34 questions were sent to all the registered panelists for a second round of the Delphi process. All the recruited panelists were included in the second round and invited to review the updated checklist. Quantitative analyses were done, which showed a high level of agreement and relevance across most questions. Descriptive analysis was done: the mean score for the relevance of the questions was more than 0.8 on all questions, indicating that at least 80% of respondents found the questions pertinent to their work and the topic at hand. Furthermore, the IQR was calculated to be 0 for all questions, highlighting a level of consensus among respondents. Based on the outcome of the Delphi study, a final checklist was outlined, with 1 more question added to make 35 questions in total (Table 1).

**Table 1.** Final draft of the Clinical AI^a^ Sociotechnical Framework (CASoF) checklist.

Stage and domain		Questions
**Planning**		
	Value proposition and utility	Have you outlined the expected impacts on patient outcomes? Have you outlined its expected impact on care provider efficiency and outcomes? Has any economic analysis been conducted for the AI system?
	Data	Have you engaged in the use of any ethical data checklist during your data collection and preparation? Have you engaged domain experts in the data preparation, cleaning, and engineering process? Have you delineated an approach to maintain data quality, integrity, and security?
	People, organization, and culture	Have you identified key stakeholders and their needs? Have you identified potential resistance or barriers within the organization? Are there strategies in place to facilitate and ensure end-user engagement in the design and development phase? Do you have a good understanding of the culture within the institution and changes that might be needed?
**Design and development**		
	Technical	Are you planning for hardware/software (EHR^b^) systems and requirements? Have you conducted a real-world evaluation of the model? Are you creating support documentation for users and management, eg, model details, explainability details, data details, metrics, manuals, etc? Have you validated clinical accuracy and reliability? Have you secured any required regulatory approval? Have you taken active steps to mitigate against biased results?
	Human-AI integration	Have you conducted a simulation with end users in real work system scenarios? Have you evaluated if the outputs are clear and understandable for the users? Have you implemented any patient and user safety measures? Have you accounted for and evaluated existing clinical workflows? Are you aligning the solution with existing protocols? Have you assessed the impact on the delivery of clinical tasks? Have you involved and tested with users? Has any resistance to the use of the AI system been identified and addressed? Are you developing strategies to ensure that the alerts from the AI system are relevant, timely, and not overwhelming, to avoid alert fatigue?
	Data	Have you tested your method on various types of data to make sure it works well in different situations? Have you planned for data drift and shift (changes in the data over time)?
**Proposed implementation**		
	People, organization, and culture	Have you ensured that this intervention aligns with the existing governance and regulatory frameworks of the organization? Have you prepared necessary training/resources for end users? Have you considered steps to help address end users’ questions and alleviate their concerns?
	Technical	Are you planning for pilot/silent tests? Are you providing user tools for continuous validation and evaluation of the system?
	Monitoring and support	Have you created a plan to evaluate the success of the implementation? Have you planned for continuous user feedback on the system? Have you planned for regular audits, reviews, and updates? Have you planned for continuous education and support for users?

a AI: artificial intelligence.

b EHR: electronic health record.

## Discussion

### Principal Findings

We introduce the CASoF checklist, which is a checklist that was developed from the results of primary studies, a literature synthesis, and a modified Delphi process that involved multiple experts and health care professionals. The CASoF, based on its sociotechnical perspective, encompasses different existing frameworks by providing a structured overview of the critical issues related to the integration, validation, and operationalization of AI in health care. The CASoF offers a high-level approach to solving the translation and adoption problems bedeviling AI systems designed for clinical settings. The CASoF can be used singly or in combination with some of the other existing frameworks in evaluating AI systems. The Diagnostic Quality Model by Lennerz et al [16] and the Clinical Explainable AI Guidelines by Jin et al [17] address diagnostic quality and explainability within medical imaging. They provide structured methodologies that could refine the CASoF by integrating rigorous quality assessments and enhancing transparency in AI tools. The strengths of these frameworks lie in their focused criteria, which could synergistically enrich the CASoF’s scope, ensuring that AI’s clinical implementation is both effective and sociotechnically sound.

At the end of the Delphi study and reviews, 35 final questions were agreed on based on the consensus from the panel members. Adjustments and rearrangements were made to the sequence of questions based on the comments made as part of the feedback during the Delphi study. This is the first checklist that addresses sociotechnical factors across the phases of the AI cycle with a general approach that is not limited to any specific condition or use case in clinical care. The checklist aims to help ensure that AI solutions for clinical use cases are better built for impact, adoption, and success.

The checklist focuses on sociotechnical factors most relevant to achieving these outcomes. Some of the comments by the respondents highlighted how the high-level design of the checklist was a reason they might not use it; however, the checklist is intentionally made high level to make it as brief and less cumbersome as possible. One of the reasons it is high level is to make it easy to apply quickly by designers, developers, AI engineers, informaticians clinicians, and health care organization managers for the needed assessments; therefore, this checklist should be considered as a form of minimum guideline in the development and implementation of AI systems meant for clinical settings.

The checklist is divided into 3 stages corresponding to the phases of the AI development cycle. The domains are drawn from the domains of the CASoF, which are “Value Proposition,” “Data,” “Human-AI Interaction,” “Organization and Culture,” “Technical,” and “Monitoring and Support” [31]. These domains are allocated to each stage based on their relevance to that stage. Some domains recur in different stages, like “Data,” “Human-AI Interaction,” “Organization and Culture,” and “Technical.” Other domains like “Value Proposition” and “Monitoring and Support” only appear in a single phase. Questions are outlined under each domain based on the stage they belong to. The number of questions varies per stage and domain.

The questions must be answered with a “Yes,” “No,” or “Partially Done.” Each stage is meant to be done before and after each corresponding phase of the development cycle, so that the development team knows what to plan for and later review what has been accomplished. The “Planning” stage addresses the decision and preparation phase of the project, which is where the groundwork is laid for the subsequent design of the system. This phase involves a value proposition assessment to determine if it ensures alignment with patients’ and end users’ benefits. It serves to help answer a “go or no go” question across the ethical, economic, and sociotechnical dimensions of the AI tool, which is part of what the “Planning” phase in the CASoF checklist is designed to support. While the Biological-Psychological, Economic, and Social checklist by Khan and Seto [32] covers the planning aspect of AI development, it does not go beyond that phase, which is a limitation in its application.

The “Design and Development” phase covers the necessary steps and factors to be considered while building the AI system, unlike the R-AI-DIOLOGY checklist, which, apart from being focused explicitly on AI systems in radiology, only addresses the technical aspects of the design and development phases [33]. The last part of the checklist helps to plan for implementation, focusing on organization, culture, and needed monitoring. The Translational Evaluation of Healthcare AI framework checklist offers an alternative to the CASoF checklist for implementation; however, its lack of sociotechnical components, such as human-AI integration, culture and organization, and monitoring and support, which are essential for adoption and maximizing utility, is a drawback [3]. The checklist’s design, development, and preimplementation aspects can also be used by payers, buyers, and decision makers to evaluate AI systems being sold or proposed to them to ensure they have been well designed and built.

Most of the existing checklists in this domain are targeted at reporting medical research carried out in AI or machine learning [34]. The CASoF checklist differs from these and other existing checklists like the Technology, Organization, and People framework–based checklist, which is focused on helping digital leaders manage adoption challenges [35]. It has no domain that addresses how the AI is designed or built, unlike the CASoF checklist. The same goes for the DECIDE-AI (Developmental and Exploratory Clinical Investigations of Decision Support Systems Driven by Artificial Intelligence) checklist, which is focused on reporting studies that involve the evaluation of AI systems during their implementation phase in the clinical setting [36]. While the CASoF checklist does not explicitly have questions that address ethical issues, there are multiple questions across different phases that raise the need to address the ethics of the data, patient outcomes, and the impact of the outputs of the AI system.

Enhancing the real-world impact of AI tools involves navigating a nuanced blend of technical and social elements. This process demands a strategic framework that guides the planning and preparation efforts throughout the AI tool’s life cycle, from its initial conceptualization to its sustained application. The CASoF checklist is designed to support designers, developers, AI engineers, informaticians, clinicians, health care organization managers, and others in planning, monitoring, and evaluating AI systems being developed or sold to them for clinical care.

### Limitations

While the primary research, literature synthesis, and Delphi technique offer a robust approach to the development of the framework and checklist for the development and integration of AI in the clinical setting, the real-world application could be more difficult and not as straightforward as the research might suggest. Therefore, there might be a need for continuous refinement of the CASoF through iterative feedback and broader engagement with more stakeholders. Future research should aim to include an even wider array of perspectives, particularly from underrepresented regions and specialties, to enhance the framework’s comprehensiveness and applicability. The framework further encounters limitations in capturing the full spectrum of technical challenges, needs, and their implications across diverse health care contexts globally. Considering these constraints, the application of the framework will benefit from synergistic application with other existing frameworks.

### Conclusion

The CASoF checklist offers an approach to bridge the gap between the technical aspects of AI and how they can be best planned to fit and work in the clinical setting, with a view to improving the impact it makes on clinical work and patient outcomes. It offers a structured strategy to mitigate challenges and obstacles in the development and implementation process. The CASoF offers an advancement over previous frameworks and approaches by holistically encapsulating the sociotechnical dimensions necessary for AI to thrive within the clinical space.

## Supplementary material

10.2196/65565 Multimedia Appendix 1 Summary of subcategories by domain for the first round of the Delphi study.

10.2196/65565 Checklist 1 PRISMA-ScR (Preferred Reporting Items for Systematic Reviews and Meta-Analyses Extension for Scoping Reviews) checklist.

## Abbreviations

AI: artificial intelligence
CASoF: Clinical Artificial Intelligence Societechnical Framework
CONSORT: Consolidated Standards of Reporting Trials
DECIDE-AI: Developmental and Exploratory Clinical Investigations of Decision Support Systems Driven by Artificial Intelligence
TRIPOD: Transparent Reporting of a Multivariable Prediction Model for Individual Prognosis or Diagnosis
