# Supplementary material for: Checklist Approach to Developing and Implementing AI in Clinical Settings: Instrument Development Study
Source: JMIRx Med. 2025 Feb 20;6:e65565. doi: 10.2196/65565 (PMC11867147; doi:10.2196/65565)
Supplement: Multimedia Appendix 1 [file xmed-v6-e65565-s001.docx]

Multimedia Appendix 1:

**Summary of sub-categories by domain for round one**

| **Stage** | **Domain** | **Number of Questions** | **Percentage of statements with Consensus** |
| --- | --- | --- | --- |
| **Planning Stage** | Value Proposition | 4 | 75% (3) |
|  | Data | 4 | 100% (4) |
|  | People | 3 | 100% (3) |
|  | Organization and Culture | 3 | 100% (3) |
| **Design Stage** | User Interface and Experience | 2 | 100% (2) |
|  | Workflow | 4 | 100% (4) |
| **Development Stage** | Technical | 3 | 100% (3) |
|  | Clinical Utility | 4 | 75% (3) |
|  | Workflow | 2 | 50% (1) |
|  | Data | 2 | 100% (2) |
|  | User Interface and Experience | 3 | 100% (3) |
| **Proposed ImplementationStage** | Organization and Culture | 1 | 100% (1) |
|  | People | 3 | 100% (3) |
|  | Technical | 3 | 100% (3) |
|  | Monitoring and Support | 4 | 100% (4) |

Note: Consensus was achieved when 80% of panelists rated a question as relevant

Total number of participants: 35
